# Author Correction: Mice lacking NF-κB1 exhibit marked DNA damage responses and more severe gastric pathology in response to intraperitoneal tamoxifen administration

**DOI:** 10.1038/s41419-019-1534-0

**Published:** 2019-04-18

**Authors:** M. D. Burkitt, J. M. Williams, T. Townsend, R. Hough, Carrie A. Duckworth, D. M. Pritchard

**Affiliations:** 1 Department of Cellular and Molecular Physiology, Institute of Translational Medicine, The Henry Wellcome Laboratory, University of Liverpool, Liverpool, UK; 2 Pathology and Pathogen Biology, Royal Veterinary College, North Mymms, UK; 3 Department of Cellular and Molecular Physiology, Institute of Translational Medicine, University of Liverpool, Liverpool, UK

**Correction to:** **Cell Death & Disease** **8**

10.1038/cddis.2017.332

published online 20 July 2017

Following the publication of this article [1], it was noted that the author list was incomplete and was missing the following author:

Carrie A. Duckworth, Department of Cellular and Molecular Physiology, Institute of Translational Medicine, University of Liverpool, Liverpool, UK

The Publisher apologizes to the authors and readers for any inconvenience caused.

1 Burkitt et al. Cell Death and Disease (2017) **8**, e2939; 10.1038/cddis.2017.332

