# Supplementary material for: Mice lacking NF-κB1 exhibit marked DNA damage responses and more severe gastric pathology in response to intraperitoneal tamoxifen administration
Source: Cell Death Dis. 2017 Jul 20;8(7):e2939–. doi: 10.1038/cddis.2017.332 (PMC5584614; doi:10.1038/cddis.2017.332)
Supplement: Supplementary Methods and Figure Legends [file cddis2017332x1.docx]

**Supplementary Methods:**

**Real-time PCR:** Groups of 3 wild-type and *Nfkb1^-/-^* mice were administered 150mg/kg tamoxifen or vehicle by IP injection. 72 hours after administration mice were culled. Stomachs were dissected and washed in two changes of PBS. Gastric mucosa was manually scraped from the omental surface of the stomach and flash frozen in liquid nitrogen. Following mechanical disruption with a TissueLyserII (Qiagen, Manchester, UK) total RNA was extracted using the Qiagen RNEasy blood and tissue kit as per manufacturer’s instructions.

qPCR was performed in a LightCycler 480 (Roche, Burgess Hill, UK) using duplex TaqMan Real-Time PCR assays. In-run reference assays were targeted to Actb (assay number: Mm00607939_s1), Atp4a was targeted with assay number Mm00444417_m1, Wnt5a with assay number Mm00437347_m1, and Erbb2 with assay number Mm00658541_m1. All assays were performed with single step reverse transcription and qPCR using TaqMan RNA-to-CT 1-Step Kit, and standard cycling conditions as per the product insert. All reagents for qPCR were sourced from ThermoFisher, Paisley, UK.

**Co-immunofluorescence:**

Standard 4µm sections were cut from formalin fixed paraffin wax embedded tissue blocks and placed on silane coated slides. Once dry, slides were exposed to Xylene and graded ethanol to rehydrate. Heat induced epitope retrieval was performed in sodium citrate buffer pH 6.0. Non-specific antibody binding was blocked by incubation in 10% chicken serum at room temperature (RT) for 45 minutes. First primary antibodies were applied for 2 hours at RT (anti-H^+^/K^+^ATPase 1:1000, anti Ki67 1:500 sources as per main materials and methods). Following wash steps secondary antibody (Chicken anti-Rabbit conjugated to Alexa Fluor 594, ThermoFisher, Cat No: A- 21442, 1:500) was applied for 45 minutes. Following further washes second primary antibody (anti- γ-H2AX, conjugated to AlexaFluor 488, ThermoFisher, Cat No: 53-9865-82, 1:500) was applied for 2 hours at RT in the dark. After final wash steps slides were coverslipped using ProLong Diamond Antifade Mountant with DAPI (ThermoFisher). Slides were imaged with a conventional fluorescence microscope with mercury arc lamp light source.

**Supplementary Figure Legends:**

**Figure S1:** Real time PCR for (A) Atp4a, (B) Wnt5a, (C) Erbb2 in WT and *Nfkb1^-/-^* mice either treated with 150mg/kg tamoxifen or vehicle for 72 hours. Statistical tests by 2-way ANOVA and Sidak’s multiple comparisons, n=3 per group. ** *p*<.01, ****p*<.001

**Figure S2:** Co-immunofluorescence for expression of H^+^/K^+^ATPase (AlexaFluor 594, red) and γ-H2AX (AlexaFluor 488, green), nuclei counterstained with DAPI. Representative images of wild-type and *Nfkb1^-/-^*  mice treated with 150mg/kg tamoxifen or vehicle for 72 hours are shown. Scale bars 100µm. Inset composite image digital magnification to show localization of staining.

**Figure S2:** Co-immunofluorescence for expression of Ki67 (AlexaFluor 594, red) and γ-H2AX (AlexaFluor 488, green), nuclei counterstained with DAPI. Representative images of wild-type and *Nfkb1^-/-^*  mice treated with 150mg/kg tamoxifen or vehicle for 72 hours are shown. Scale bars 100µm. Inset composite image digital magnification to show localization of staining.
