# Supplementary figures and images for: Mice lacking NF-κB1 exhibit marked DNA damage responses and more severe gastric pathology in response to intraperitoneal tamoxifen administration
Source: Cell Death Dis. 2017 Jul 20;8(7):e2939–. doi: 10.1038/cddis.2017.332 (PMC5584614; doi:10.1038/cddis.2017.332)

**A****ATP4A**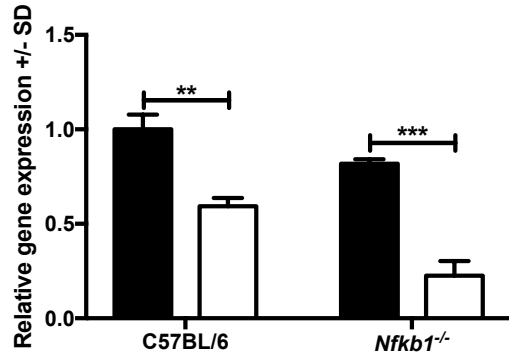**B****WNT5A**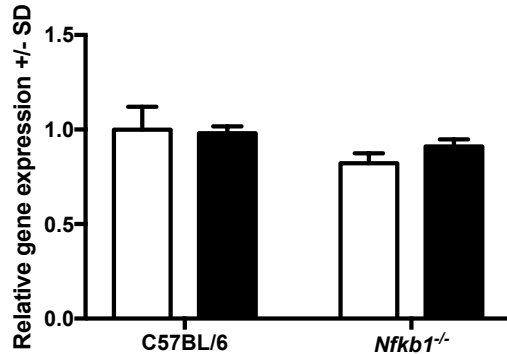**C****ErbB2**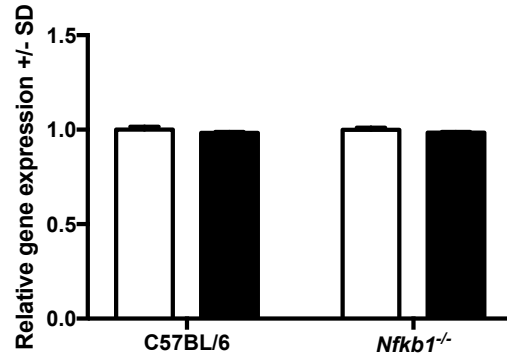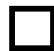

Vehicle

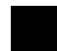

Tamoxifen

Supplement: Supplementary Figure 1 [file cddis2017332x2.pdf]

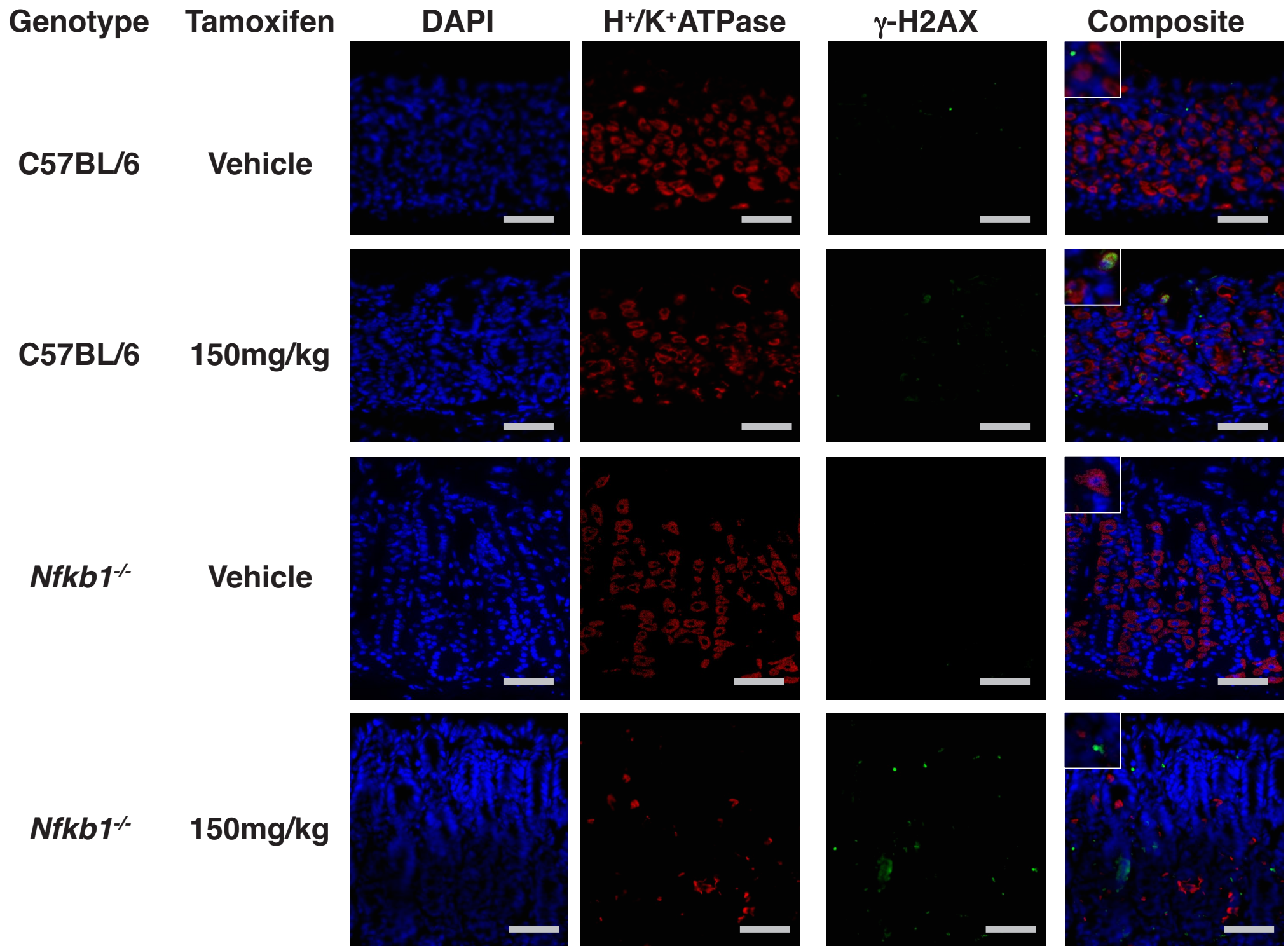

Supplement: Supplementary Figure 2 [file cddis2017332x3.pdf]

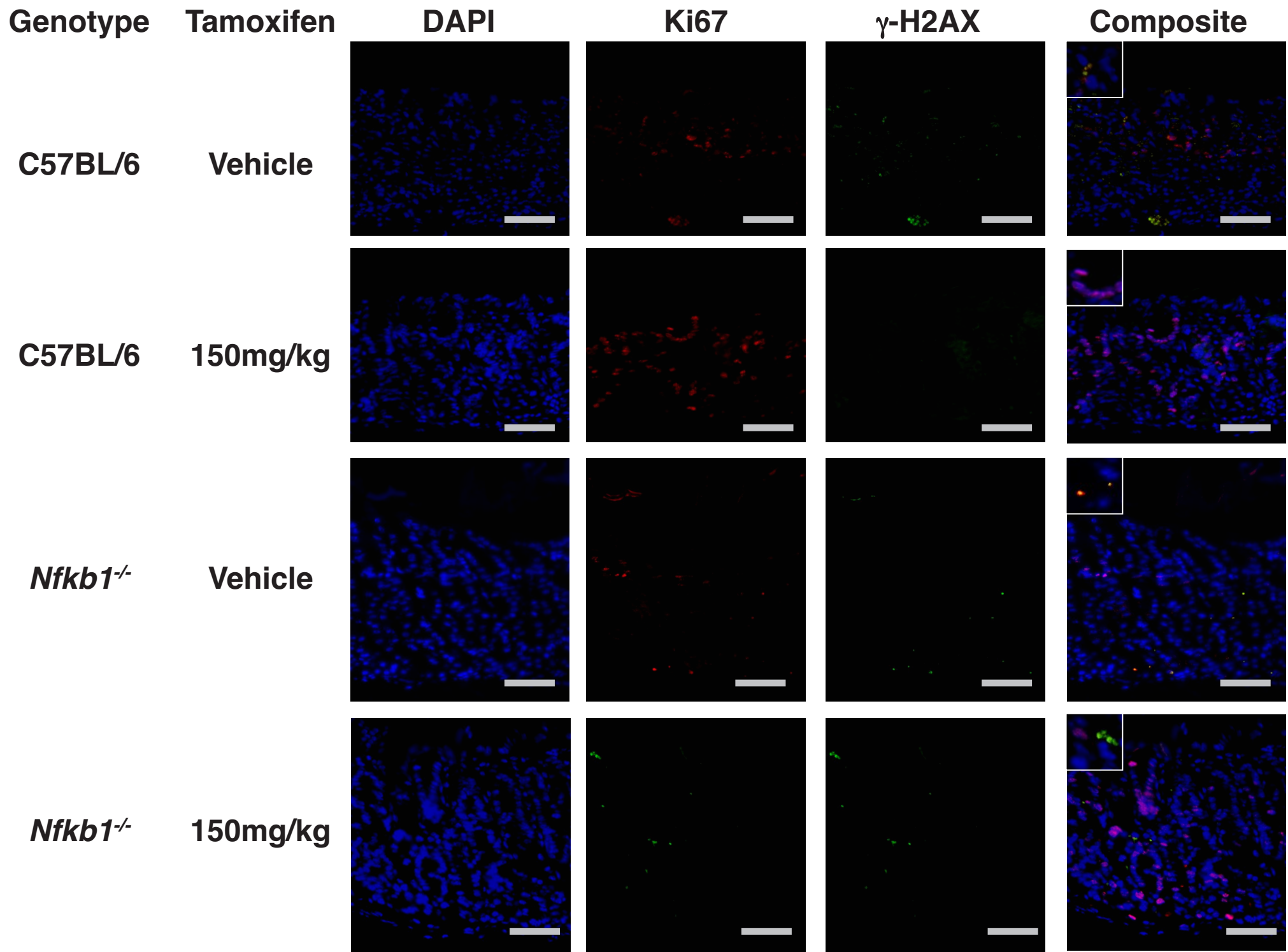

Supplement: Supplementary Figure 3 [file cddis2017332x4.pdf]
